# Implicit theories of anxious and regulated shyness in American and Chinese children

**DOI:** 10.3389/fpsyg.2026.1766694

**Published:** 2026-03-17

**Authors:** Yiyuan Xu, Luyen Lin

**Affiliations:** Department of Psychology, University of Hawaii at Manoa, Honolulu, HI, United States

**Keywords:** anxious shyness, Chinese, culture, implicit theories, regulated shyness

## Abstract

**Introduction:**

Emerging evidence suggests that cultural differences in interpersonal perception may influence how shy children adjust across cultural contexts. This study examined whether implicit theories of shyness mediate cross-cultural differences in children's relationships with shy peers and whether this mediating effect differs by the form of shyness.

**Methods:**

Participants were 94 American children from Honolulu, USA (48 girls; M age = 10.24 years) and 106 Chinese children from Shanghai, PRC (52 girls; M age = 10.28 years). Children were randomly assigned to read vignettes depicting either anxious shyness or regulated shyness. They then completed measures assessing their implicit theories of the depicted form of shyness and their relationships with peers showing that form of shyness.

**Results:**

Stronger entity theories of shyness were associated with poorer relationships with shy peers, indicating that implicit theories may play a similar role in explaining individual differences in shy children's adjustment across both cultural groups. American children endorsed stronger entity theories of shyness than Chinese children, which partly mediated their poorer relationships with shy peers. This mediating effect was stronger for children who read the anxious shyness vignette than for those who read the regulated shyness vignette.

**Discussion:**

These findings highlight the importance of implicit theories of shyness in understanding both cultural similarities and cultural differences in children's relationships with shy peers.

## Introduction

Childhood shyness has been of considerable interest to cultural and cross-cultural researchers, particularly in comparisons between Chinese and North American children (Chen, 2018). Research conducted in Western contexts has tended to associate shyness with adjustment and relationship problems (Rubin and Coplan, 2010; Schmidt and Poole, 2020). By contrast, studies of Chinese children have produced mixed findings, depending on how shyness is conceptualized (Xu et al., 2020) and when and where the studies were conducted (Chen, 2018; Xu and Suh, 2022). Nevertheless, recent research has shown that shyness remains more strongly related to adjustment problems, such as social anxiety, in North American children than in Chinese children (e.g., Kong et al., 2023).

Despite considerable progress, most studies have relied on *post-hoc* interpretations of cross-cultural differences without directly examining specific explanatory factors at the group level, particularly those related to interpersonal perception, which are largely shaped by cultural values and socialization. More recently, researchers have begun to investigate how cultural variations in children's implicit theories, or the ways they interpret and react to their own or others' attributes (Chiu et al., 1997), may help explain cross-cultural differences in the perception of, and relationships with, shy children (Zhang and Xu, 2019).

Implicit theories can be conceptualized along a continuum with two contrasting views at opposite ends (Erdley and Dweck, 1993; Erdley et al., 1997; Levy and Dweck, 1999): an entity theory, which construes traits or behaviors as fixed and immutable, often leading to global, rigid, and enduring judgments of others based on limited information; and an incremental theory, which emphasizes the malleable nature of abilities and personalities across time and situations, typically fostering more flexible interpersonal judgments. There is preliminary evidence that American children on average reported stronger entity theories (or less incremental theories) of shyness than Chinese children, and were more likely to view shyness as a stable and immutable trait, which partly explained why they reported poorer relationship with shy peers (Zhang and Xu, 2019).

One of the key limitations of Zhang and Xu (2019) was their conceptualization of shyness as a unidimensional construct. However, growing evidence suggests that childhood shyness is a multifaceted phenomenon (Xu et al., 2020). Distinct forms of shyness, such as anxious shyness and regulated shyness, have been identified and differentiated in both Western and non-Western contexts (Kong et al., 2023; Özdemir et al., 2015; Xu et al., 2007; Xu and Farver, 2009; Xu et al., 2014; Xu and Krieg, 2014; Xu and Zhang, 2025; Xu et al., 2008). Therefore, the purpose of the current study was to investigate how American and Chinese children differed in their implicit theories of shyness, and how such differences may help explain their varying relationship with shy peers and vary as a function of form of shyness.

### Childhood shyness in the North American and Chinese contexts

Childhood shyness has attracted considerable interest due to its close relation to important developmental milestones, such as gaining social acceptance and forming friendships with peers (Rubin and Coplan, 2010). Abundant research has shown that childhood shyness is consistently associated with poor psychosocial adjustment and peer relationships in North American contexts (Rubin and Coplan, 2010). However, findings involving children from other cultural settings, particularly Chinese culture, have been less clear-cut. For instance, in a series of studies on shyness-sensitivity, defined as the tendency to be shy, sad, and easily hurt, Chen and colleagues found that shyness-sensitivity was related to better social functioning and peer relationships among Chinese children compared to Canadian children. They interpreted these results as reflecting more positive perceptions and evaluations of shy-sensitive children by Chinese peers than by Canadian peers (Chen et al., 1992, 1995). In contrast, in a comparison of three different cohorts in the 1990s and 2000s, Chen et al. (2005) demonstrated that shyness-sensitivity became increasingly associated with negative outcomes among more recent cohorts of Chinese children in the 2000s. These cohorts were presumed to have experienced greater cultural and societal transformations and to be more susceptible to Western values emphasizing assertiveness and independence. Chen et al. (2005) argued that their findings were consistent with changing social perceptions and evaluations of shy-sensitive children in contemporary Chinese society. However, despite growing interest in understanding cross-cultural and cross-cohort differences in the outcomes associated with childhood shyness, few studies have directly examined whether such differences are linked to variations or shifts in social perceptions of shyness, as implied in *post hoc* interpretations of prior findings.

### Individual differences in implicit theories of shyness

Research on interpersonal perception suggests that implicit theories shared by lay people, i.e., entity and incremental theories mentioned above, represent an important way how they interpret and react to social situations (Chiu et al., 1997). Studies of within-cultural *individual differences* in implicit theories have shown that children with stronger entity views of others are more judgmental, more likely to label others, more likely to make extreme trait ratings, and less willing to interact with others with traits that they believe are undesirable (Giles and Heyman, 2004; Levy and Dweck, 1999). In addition, they also tend to develop stereotypes and overgeneralization of behavioral characteristics (Erdley et al., 1997; Levy and Dweck, 1999).

Beer (2002) extended implicit self-theories to understand within-cultural *individual differences* in adult shyness. Specifically, she found that those with stronger entity beliefs that their own shyness was fixed and would not change over time and across situations even with effort, were less likely to view social situations as a learning opportunity and to approach social settings, were more likely to use strategies aimed at avoiding social interaction, and suffered more negative consequences of their shyness. These results demonstrated that individuals with varying implicit theories of their own shyness may experience different adjustment outcomes.

### Cultural group differences in implicit theories of shyness

Numerous cross-cultural works have demonstrated that culture plays an important role in forming an interpretative framework within which traits and behavior are evaluated and implicit theories are developed in accordance with predominant cultural values and beliefs (Choi et al., 1999; Heine and Buchtel, 2009; Lockhart et al., 2008). Studies have demonstrated that American parents tend to attribute children's failures to a lack of ability, whereas Japanese mothers view academic success as more the result of effort than innate ability (Heine, 2001, 2005; Hess et al., 1987; Holloway, 1988; Sato et al., 2004). Lockhart et al. (2008) compared American and Japanese children's responses to vignettes that described hypothetical characters with various physical (e.g., short statue) and psychological traits (e.g., unkind), and found that Japanese children were more optimistic about others' negative traits changing toward the positive than American children, and were more likely to attribute differences in trait expression to effort than to innateness; they seemed to hold more of an incremental view than their American counterparts.

Building on Lockhart et al. (2008) and Beer (2002), Zhang and Xu (2019) examined between-cultural *group differences* in implicit theories about their peers' shyness between American and Chinese children, and how these between-cultural *group differences* in implicit theories may be related to between-cultural *group differences* in relationships with shy peers. They found that on average American children reported stronger entity views of shyness than Chinese children, suggesting that American children were more likely to believe that shyness was relatively stable and immutable. In addition, relatively stronger entity theories among American children partly mediated why they reported worse relationship with shy peers than their Chinese counterparts, providing preliminary support for the important role between-cultural *group differences* in implicit theories may play in understanding varying adjustment outcomes and social relationships associated with childhood shyness across cultural contexts.

### Anxious and regulated shyness

Zhang and Xu (2019) did not distinguish different forms of shyness and associated entity and incremental theories, a limitation the current study aimed to address. Studies conducted with both non-Western (Özdemir et al., 2015; Xu et al., 2007, 2009, 2014; Xu and Zhang, 2025) and Western contexts (Kong et al., 2023; Xu and Krieg, 2014) have similarly differentiated anxious and regulated shyness in children, along with the varying outcomes associated with them. Anxious shyness is defined as social inhibition and wariness toward familiar peers or authority figures, possibly due to concerns about negative social evaluation, whereas regulated shyness refers to non-assertive, unassuming, and polite behavior, particularly in conspicuous and potentially conflictual situations (Asendorpf, 2009; Xu et al., 2007, 2009, 2020).

Regardless of cultural contexts, children appeared to hold more positive views of regulated than anxious shyness, which may influence their implicit theories of these two forms of shyness (Xu et al., 2020). Studies have found that children, especially younger ones, are more likely to believe in effort as a way of changing traits or behavior toward a positive direction, a phenomenon named *positivity bias* (Boseovski, 2010). Positivity bias seems particularly salient in implicit theories of others' personality and emerges as early as 3 years of age (Boseovski and Lee, 2006), and become increasingly common in middle childhood (Benenson and Dweck, 1986; Heyman and Giles, 2004). Consequently, Children's beliefs in stability of traits seem to vary in accordance to perceived valence of traits, i.e., an entity view may be stronger for more favorable attributes (Boseovski, 2010; Heyman and Giles, 2004). Lockhart et al. (2002) found that compared to negative traits, positive traits were judged by children as more stable and more likely related to “nature” than “nurture” explanations. Heyman and Dweck (1998) showed that children of 7–8 years held stronger stability beliefs in positive sociomoral traits, or “goodness,” than negative sociomoral traits or “badness.”

Prior studies have demonstrated that while anxious shyness was viewed negatively across cultural contexts, regulated shyness was considered more favorably in the Chinese than American cultures, possibly due to stronger functional roles regulated shyness may play in maintaining social harmony in the Chinese than American cultural context (Xu and Krieg, 2014; Xu et al., 2020). However, regulated shyness appears to be a more preferable attribute than anxious shyness in both North American and Chinese contexts (Kong et al., 2023; Xu et al., 2020). Therefore, given the role perceived valence may play in stability beliefs, more favorable views of regulated than anxious shyness may influence the extent to which children hold entity or incremental theories of these two forms of shyness, regardless of cultural context. That is, both American and Chinese children may hold stronger entity theories of regulated shyness than those of anxious shyness.

### The current study

The current study sought to address two research questions: (1) how implicit theories of shyness and relationship with shy peers may differ between cultures (American or Chinese) and depend on form of shyness (regulated or anxious shyness); and (2) how cultural differences in implicit theories of anxious (or regulated) shyness may help explain (mediate) varying relationships with anxiously (or regulated) shy peers between American and Chinese children, and how this mediating effect might be moderated by form of shyness.

Regarding the first research question, after controlling for age, gender, and children's own anxious and regulated shyness, we expected that compared to their Chinese counterparts, American children would on average endorsed more entity theories and have poorer relationship with their shy peers. Given the positivity bias found in previous studies, we expected that when compared to children who read the vignette of anxious shyness, those who read the vignette of regulated shyness, would report stronger entity theories of shyness, and better relationship with shy peers.

For the second research question, after controlling for age, gender, and children's own anxious and regulated shyness, we expected that stronger entity views of anxious (or regulated) shyness among American than Chinese children would at least partly explain (mediate) their poorer relationship with anxiously (or regulated) shy peers. In addition, we explored whether the strength of mediation would vary depending on whether children read the vignette of regulated or anxious shyness, but did not specify any hypothesis.

## Method

### Participants

The sample consisted of 94 American children from Honolulu, Hawai'i (48 females and 46 males; *M*age = 10.24, *SD* = 0.75) and 106 Chinese children from Shanghai, PRC (52 females and 54 males; *M*age = 10.28, *SD* = 0.41). We focused on this age group because beliefs about stability/malleability of traits seem to become relatively coherent at around 9–10 years (Gelman et al., 2007). Chinese children were recruited from an elementary school in Shanghai, PRC. The elementary school had four fourth- and fifth-grade classes with about 40 students per class. Two fourth grade and two fifth grade classes participated. The consent rate was about 66% and all children are ethnically *Han*. To match the Chinese sample, we recruited fourth and fifth grade American children from local communities in Honolulu through flyers and word of mouth. The participating American children were all born in the U.S. and comprised of 39.4% Asian American, 35.1% Multiracial, 13.8% European American, and 11.7% Native Hawaiian and Pacific islander children, which reflected the racial diversity of Hawai'i in general. The consent rate was about 60%.

### Measures and procedure

Data were collected using both child interviews and parental reports. The procedure of child interview, as well as the questions on implicit theories and relationship with shy peers were adapted from Zhang and Xu (2019), whereas parents reported their children's anxious and regulated shyness using the Chinese Shyness Scale (CSS; Xu et al., 2007, 2009; Kong et al., 2023). Prior to the formal interviews, pilot interviews were conducted with 10 children from each cultural group to make sure that children felt comfortable with the procedure and understood the questions.

A 2 (cultural group: American or Chinese) X 2 (shyness condition: anxious or regulated shyness vignette) quasi-experimental design was used. Specifically, for both cultural groups, children were randomly assigned to one of the two conditions during which a trained research assistant, matched to the child's gender, read a short vignette of either anxious shyness or regulated shyness and then asked about children's implicit theories of shyness and relationship with shy peers (see details below). To minimize the order effect, half of the children received the questions on implicit theories first, whereas the other half were asked about their relationships with shy peers first. In addition, the order of the questions on implicit theories and relationship with shy peers were randomized. The interviews were conducted in a quiet location familiar to the child, such as school cafeteria or meeting room.

**Implicit theories of anxious (or regulated) shyness**. To assess children's implicit theories of anxious (or regulated) shyness, a trained research assistant read aloud a short vignette of either anxious shyness (*n* = 47 for American sample and *n* = 53 for Chinese sample) or regulated shyness (*n* = 47 for American sample and *n* = 53 for Chinese sample), adapted from the anxious shyness and regulated shyness subscales of the Chinese Shyness Scale (CSS; Xu et al., 2007, 2009; Kong et al., 2023):

“*(Anxious shyness) There are some shy kids at school. They are timid, isolated, afraid of playing with peers, and often nervous when speaking in front of others*.”

“*(Regulated shyness) There are some shy kids at school. They are modest, polite, do not like showing off, and often avoid conflict with others*.”

Following the description of either anxious or regulated shyness, half of the children received questions on implicit theories first, whereas the other half received the questions on relationship with shy peers first.

Research assistants asked five questions related to implicit theories of anxious (or regulated) shyness, adapted from Beer (2002) and Zhang and Xu (2019): “*Do you think their shyness is something about them that they can't change very much?*” “*Do you think nothing they do will change their shyness?*” “*Do you think they can change their shyness if they want to (reverse coded)?* “*Do you think they will always be that way even when they grow up?*” “*Do you think their shyness is not fixed, but changes over time (reverse coded)?*”

Children indicated on a five-point scale ranging from 1 (very strongly disagree) to 5 (very strongly agree) the extent to which they believed each of the five statements, with higher average scores indicating stronger entity theories and lower average scores indicating stronger incremental theories (Both Cronbach's alphas and McDonald's omegas were 0.90 for the American sample and 0.88 for the Chinese sample). This approach is consistent with the evidence that individuals who disagree with entity theory statements tend to agree with statements consistent with an incremental theory (Beer, 2002; Erdley and Dweck, 1993; Erdley et al., 1997; Levy and Dweck, 1999; Rudolph, 2010).

To examine whether the properties of the implicit theories measure were invariant between American and Chinese children, we conducted a series of multi-group confirmatory factor analyses using the alignment optimization method (Asparouhov and Muthén, 2014, 2023). Specifically, configural invariance is first evaluated as the baseline model, followed by estimation of item loadings and intercepts by minimizing the overall non-invariance, and calculation of latent factor means without requiring strict scalar invariance that is difficult to achieve (Asparouhov and Muthén, 2014). We applied the fixed option (due to the limited number of groups) and restrictive rule of thumb suggested by Asparouhov and Muthén (2014), where no more than 25% of parameters should be non-invariant. The results of alignment method (Asparouhov and Muthén, 2014) supported approximate measurement invariance between cultural groups (see Supplementary material).

**Relationship with anxiously (or regulated) shy peers**. Research assistants asked three questions on relationship with anxiously (or regulated) shy peers, adapted from Zhang and Xu (2019). Specifically, children were asked nominate one anxiously (or regulated) shy peer they are familiar with, depending on the experimental condition they were assigned to: “*Now think about a child you know who is like them [children descried in the anxious or regulated shyness vignette]*,” and then responded to three questions on three-point scales regarding their relationship with this anxiously (or regulated) shy peer. These questions included whether they were playmates with this peer, “*how often do you play with this child*? *not at all (1), sometimes (2), or a lot (3)*;” whether they were friends with this peer, “w*ould you consider this child: not your friend (1), one of your friends (2), or one of your best friends (3)?*” and the extent to which they liked this peer, “*how do you like this child? Not at all (1), somewhat (2), or a lot (3)*.”

**Parents' ratings of children's anxious and regulated shyness**. Parents were asked to complete and return the two subscales of the Chinese Shyness Scale (CSS; Xu et al., 2007, 2009) along with their consent forms: anxious shyness (5 items, e.g., “afraid to join or approach peer play groups”) and regulated shyness (5 items, e.g., “Does not show off”). Average scores were calculated for each subscale (1 = not at all true; 5 = very true). The CSS has been validated in both elementary school (Xu et al., 2007, 2009) and preschool children (Kong et al., 2023), and across different informants, including peers (Xu et al., 2007), teachers (Xu et al., 2009), and parents (Kong et al., 2023). Both Cronbach's alphas and McDonald's omegas for anxious and regulated shyness subscales were 0.89 and 0.87 for American children, and 0.94 and 0.88 for Chinese children. The results of alignment method (Asparouhov and Muthén, 2014) supported approximate measurement invariance for anxious and regulated shyness between cultural groups (see Supplementary material).

## Results

### Preliminary analyses

**Descriptive statistics and correlations among variables**. Descriptive statistics and correlation among variables were summarized in Tables 1, 2. The correlations among variables demonstrated mostly cultural similarities; the inter-correlations among the variables on implicit theories and relationship with anxiously (or regulated) shy peers were similar for both cultural groups. Stronger entity theories of anxiously (or regulated) shyness were negatively associated with the three variables on relationship with anxiously (or regulated) shy peers (i.e., playmates, friends, and liking). Children who read the description of regulated shyness, reported stronger entity theories and liked their peers more than children who read the description of anxious shyness, but did not play more with, or were more likely to be friends with regulated shy peers.

**Table 1:** Descriptive statistics for American and Chinese children.

**Variable**	American (***n*** = 94)		Chinese (***n*** = 106)	
	**Regulated shyness condition (*****n*** = **47)**	**Anxious shyness condition (*****n*** = **53)**	**Regulated shyness condition (*****n*** = **47)**	**Anxious shyness condition (*****n*** = **53)**
	**Mean (** ***SD*** **)**	**Mean (** ***SD*** **)**	**Mean (** ***SD*** **)**	**Mean (** ***SD*** **)**
Entity theories of shyness	3.12 (0.69)	2.40 (0.81)	2.32 (0.72)	1.91 (0.69)
Playmates with shy peers	1.53 (0.75)	1.45 (0.65)	2.04 (0.59)	1.83 (0.64)
Friends with shy peers	1.45 (0.50)	1.40 (0.58)	1.89 (0.64)	1.79 (0.72)
Liking of shy peers	1.77 (0.56)	1.51 (0.59)	1.92 (0.43)	1.68 (0.51)
Anxious shyness	2.09 (0.99)	2.22 (0.96)	2.31 (1.06)	2.14 (1.03)
Regulated shyness	2.54 (0.89)	2.52 (0.90)	3.07 (1.30)	3.10 (1.23)
Age	10.23 (0.69)	10.24 (0.82)	10.27 (0.47)	10.29 (0.35)

Self-reported entity theories of shyness, parents' ratings of anxious shyness, and parents' ratings of regulated shyness were rated on a five-point scale; playmates with shy peers, friends with shy peers, and liking of shy peers were rated by children on a three-point scale.

**Table 2:** Correlations among variables (*n* = 94 for American, *n* = 106 for Chinese).

**Variable**	**1**	**2**	**3**	**4**	**5**	**6**	**7**	**8**	**9**
1. Entity theories of shyness	1.00	−0.31^**^	−0.31^**^	−0.26^**^	0.17	−0.01	−0.02	0.02	−0.28^**^
2. Playmates with shy peers	−0.25^*^	1.00	0.34^**^	0.37^**^	0.06	−0.02	0.17	−0.07	−0.17
3. Friends with shy peers	−0.22^*^	0.27^**^	1.00	0.37^**^	0.06	−0.08	0.08	0.02	−0.07
4. Liking of shy peers	−0.24^**^	0.33^**^	0.39^**^	1.00	0.15	−0.07	0.06	0.07	−0.25^**^
5. Regulated shyness	0.02	−0.01	−0.02	0.14	1.00	−0.02	0.11	0.02	0.01
6. Anxious shyness	0.06	−0.08	−0.02	−0.15	0.13	1.00	−0.10	0.05	−0.08
7. Age	0.17	−0.05	−0.30^**^	−0.22^*^	0.07	−0.03	1.00	0.11	0.03
8. Gender	−0.14	−0.02	−0.06	0.06	0.10	−0.08	0.11	1.00	–
9. Shyness condition	−0.43^**^	−0.06	−0.04	−0.22^*^	−0.01	0.07	0.00	–	1.00

^*^p < 0.05; ^**^p < 0.01.

Correlations among the diagonal were for Chinese children; Gender (0 = female, 1 = male); Shyness condition (0 = description of regulated shyness, 1 = description of anxious shyness).

Regardless of cultural group, parents' ratings of children's own anxious and regulated shyness were not related to children's implicit theories and the three variables on relationship with anxiously (or regulated) shy peers. Gender (1 = boy, 0 = girl) was not correlated with any variables. Age was negatively correlated with making friends with anxiously (or regulated) shy peers and liking of anxiously (or regulated) shy peers among American but not Chinese children.

**Ethnic differences among American children**. Due to the insufficient subgroup sizes of European Americans (*n* = 13) and “others” (Native Hawaiians and Pacific Islanders, *n* = 11), we were only able to explored ethnic differences between Asian Americans (*n* = 37) and multiracial children (*n* = 33) on all the variables using *t*-tests. No group differences were found: *t*s ranged from −1.08 to 1.24, *p*s ranged from 0.22 to 0.89; Cohen's *d*s ranged from 0.03 to 0.30.

### Cultural differences in implicit theories of shyness and relationship with shy peers

2 (American or Chinese) X 2 (anxious or regulated shyness) ANCOVAs were conducted to examine how children's implicit theories of shyness differed across culture and experimental shyness condition, after controlling for age, gender, and children's own anxious and regulated shyness. The results of ANCOVAs are summarized in Table 3. Consistent with our expectation, American children (*M* = 2.77, *SD* = 0.83) reported stronger entity theories of shyness [*F*_(1, 190)_ = 41.98, *p* < 0.001, partial eta squared = 0.18] than did Chinese children (*M* = 2.12, *SD* = 0.73). Also in line with our expectation, children who read the vignette of regulated shyness (*M* = 2.70, *SD* = 0.81), reported stronger entity theories of shyness [*F*_(1, 190)_ = 30.10, *p* < 0.001, partial eta squared = 0.14] than did children who read the vignette of anxious shyness (*M* = 2.14, *SD* = 0.78). The culture X shyness condition interaction was not significant [*F*_(1, 190)_ = 2.25, *p* = 0.14, partial eta squared = 0.01].

**Table 3:** Analyses of covariances (ANCOVAs).

**Effect**	***df***	***F* test**	**Partial η^2^**
**Entity theories of shyness**			
Culture	1	41.98^**^	0.18
Shyness condition	1	31.10^**^	0.14
Interaction	1	2.25	0.01
**Playmates with shy peers**			
Culture	1	19.33^**^	0.09
Shyness condition	1	2.10	0.01
Interaction	1	0.67	0.00
**Friends with shy peers**			
Culture	1	19.42^**^	0.09
Shyness condition	1	0.44	0.00
Interaction	1	0.21	0.00
**Liking of shy peers**			
Culture	1	2.72	0.01
Shyness condition	1	10.87^**^	0.05
Interaction	1	0.04	0.00

^*^p < 0.05; ^**^p < 0.01.

ANCOVAs were conducted after controlling for age, gender, and children's own anxious shyness and regulated shyness; Culture (American or Chinese); Shyness condition (anxious or regulated shyness vignette); Interaction (Culture X shyness condition); Entity theories of anxious (or regulated) shyness were rated on a five-point scale; playmates with anxiously (or regulated) shy peers, friends with anxiously (or regulated) shy peers, and liking of anxiously (or regulated) shy peers were rated on a three-point scale.

2 (American or Chinese) X 2 (anxious or regulated shyness) ANCOVAs were also conducted to examine how children's relationship with anxiously (or regulated) shy peers differed across culture and shyness condition after controlling for age, gender, and children's own shyness. Consistent with our expectation, compared to American children (*M*s = 1.50, 1.43; *SD*s = 0.70, 0.54), Chinese children (*M*s = 1.93, 1.84; *SD*s = 0.62, 0.68) were more likely to be playmates [*F*_(1, 190)_ = 19.33, *p* < 0.001, partial eta squared = 0.09] or friends with shy peers [*F*_(1, 190)_ = 19.42, *p* < 0.001, partial eta squared = 0.09], regardless of shyness condition. Inconsistent with our expectation, Chinese children did not like their shy peers more than their American counterparts [*F*_(1, 190)_ = 2.72, *p* = 0.10, partial eta squared = 0.01] when the two shyness conditions were combined in the analyses.

In line with our prediction, compared to children who read the vignette of anxious shyness (*M* = 1.61, *SD* = 0.55), those who read the vignette of regulated shyness (*M* = 1.85, *SD* = 0.50), liked their shy peers more [*F*_(1, 190)_ = 10.87, *p* = 0.001, partial eta squared = 0.05]. However, contrary to what we expected, they were *not* more likely to play with [*F*_(1, 190)_ = 2.10, *p* = 0.15, partial eta squared = 0.01] or be friends with shy peers [*F*_(1, 190)_ = 0.44, *p* = 0.51, partial eta squared = 0.00]. The culture X shyness condition interactions were not significant: [*F*_(1, 190)_ = 0.67, 0.21, 0.04, *ps* = 0.41, 0.65, 0.84, partial eta squared = 0.00, 0.00, 0.00].

### Cultural differences in implicit theories of shyness in relation to cultural differences in children's relationship with shy peers

**Figure 1:**
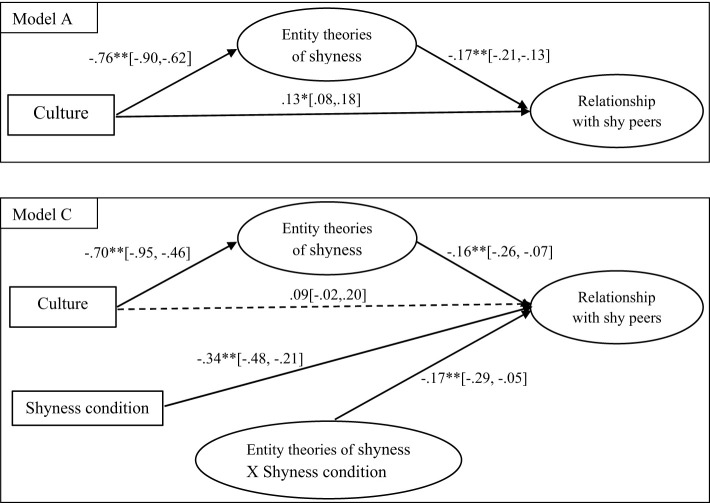
Final mediation model (Model A) and moderated mediation model (Model C). *p < 0.05, **p < 0.01; unstandardized path coefficients and their 95% confidence intervals were reported; Model A = partial mediation model (with children's own regulated shyness as the control variable Model C = moderated mediation model); Culture (0 = American, 1 = Chinese); Shyness condition (0 = regulated shyness, 1 = anxious shyness).

Structural equation modeling was conducted to first examine whether cultural differences in entity theories of (anxious or regulated) shyness helped explain (mediated) children's relationship with anxiously (or regulated) shy peers, after controlling for age, gender, and children's own anxious and regulated shyness. Without differentiating the two experimental shyness conditions, we first compared a partial mediation (Model A) and a full mediation model (Model B), with or without the direct effect of culture on relationship with shy peers. Specifically, culture (dummy coded; 0 = American, 1 = Chinese) was specified as the predictor, entity theories of shyness as the latent mediator (with five item scores as the indicators), and relationship with shy peers (with three indicators: playmates with shy peers, friends with shy peers, and liking of shy peers) as the latent outcome construct. Control variables were allowed to have effects on both the mediator and outcome, but we only found significant relation between children's own regulated shyness and relationship with shy peers. Thus, age, gender, and children's anxious shyness were removed from the final model A and B. Both Model A [$\chi_{\left(31\right)}^{2}$ = 49.49, *p* = 0.02; CFI = 0.97; TLI = 0.96; RMSEA = 0.06, SRMR = 0.04] and Model B [$\chi_{\left(32\right)}^{2}$ = 54.29, *p* = 0.01; CFI = 0.97; TLI = 0.96; RMSEA = 0.06, SRMR = 0.04] fit the data reasonably well, providing support for the mediating role of entity theories of shyness in explaining cultural group differences in relationship with shy peers. However, the partial mediation Model A (see Figure 1) with direct effect of culture on children's relationship with peers was retained due to its better fit: Δ$\chi_{\left(1\right)}^{2}$ = 4.80, *p* = 0.03.

### Exploring the moderating role of form of shyness

The moderating role of form of shyness was explored in Model C via the interaction between shyness condition (0 = regulated shyness, 1 = anxious shyness) and the latent mediator (implicit theories of shyness). Specifically, this interaction was added to Model A and allowed to be related to the latent outcome variable: relationship with anxiously (or regulated) shy peers (see Model C in Figure 1). We used the Latent Moderated Structural Equations (LMS) approach (Klein and Moosbrugger, 2000), implemented in Mplus by Stride and his colleagues (Stride et al., 2015). The LMS approach uses a mixture model to approximate the resulting non-normal distribution with full-information maximum likelihood estimation (FIML). However, due to the use of numerical integration, Chi-square and related fit statistics are not available.

As shown in Model C (Figure 1), there was a significant path of Shyness condition → Relationship with shy peers, indicating that children's peer relationship differed depending whether they read the vignette of regulated or anxious shyness. While the direct effect of Culture → Relationship with shy peers was no longer significant, the mediating path, or the indirect effect of Culture → Entity theories of shyness → Relationship with shy peers remain significant. This mediating path was moderated by the interaction of Entity Theories of Shyness X Shyness Condition, with an estimated Index of Moderated Mediating (IMM) effect of 0.12 (*p* = 0.01; 95% CI [0.03, 0.21]; Hayes, 2015; Stride et al., 2015). Specifically, the indirect (mediating) effect of Culture → Entity theories of shyness → Relationship with shy peers was stronger when children read the vignette of anxious (indirect effect = 0.24; *p* < 0.01; 95% CI [0.20, 0.35]) than regulated shyness (indirect effect = 0.12, *p* < 0.01; 95% CI [0.04, 0.19]).

Finally, given that the participants of American children included several ethnic subgroups, we conducted sensitivity analyses (e.g., repeating analyses while excluding one subgroup at a time) to demonstrate the robustness of the moderated mediating effect (see Supplementary material).

## Discussion

Numerous efforts have been made to investigate cultural similarities and differences in the adjustment of shy children in North American and Chinese contexts. However, only recently have researchers begun to directly examine group-level explanatory factors, particularly those related to cultural variations in interpersonal perception, such as implicit theories of shyness that likely develop in accordance with predominant cultural values and beliefs (Zhang and Xu, 2019). Building on prior work on within-cultural individual differences in implicit theories, we extend the theoretical framework to explore how between-cultural group differences in implicit theories of others' shyness, a potential group-level mediator, may help explain cultural differences in children's relationships with shy peers, as well as how this mediating effect may be moderated by the form of shyness.

### Within-cultural individual differences in implicit theories of shyness and relationship with shy peers

Individual differences in implicit theories of others' attributes have been of considerable interest in prior research on children's social perception and peer relationships. For instance, in a series of studies, Yeager and colleagues showed that stronger entity theories, or beliefs about the unchangeable and immutable nature of others' attributes, are related to more rigid and unfavorable perceptions and evaluations of others. They further demonstrated that changes in implicit theories of others' characteristics may causally influence how adolescents interact with peers (Yeager and Miu, 2011; Yeager et al., 2013a,b). Although the primary focus of the present study was on between-cultural group differences, it is important to note that our results revealed remarkable cultural similarities in the intercorrelations between individual differences in implicit theories of shyness and individual differences in relationships with shy peers. Specifically, in both cultural contexts, stronger entity theories of anxious (or regulated) shyness were negatively associated with the frequency of playing with anxiously (or regulated) shy peers, forming friendships with anxiously (or regulated) shy peers, and liking anxiously (or regulated) shy peers. Moreover, entity theories of anxious (or regulated) shyness were unrelated to gender or to children's own levels of anxious or regulated shyness in either cultural group. Thus, our findings align with Yeager et al. (2013a,b), providing support for the idea that implicit theories may play similar functional roles in shaping children's relationships with their anxiously (or regulated) shy peers across cultures.

### Between-cultural comparisons in implicit theories of shyness and relationship with shy peers

Prior cross-cultural research has shown that North American parents and children tend to attribute academic and social success or failure to innate traits or abilities (Heine, 2001, 2005; Li, 2004) and to hold stronger stability beliefs about individual attributes than their Asian counterparts (Lockhart et al., 2008). Zhang and Xu (2019) compared implicit theories of shyness, without differentiating between its varying forms, between American and Chinese children, and found higher ratings of entity beliefs about shyness among American than Chinese children. Consistent with this line of work, the significant main effect of cultural group and the lack of interaction between cultural group and shyness condition in the present study indicate that American children, on average, endorsed stronger entity views about shyness than Chinese children, suggesting that American children are more likely to view shyness as relatively stable and immutable, even when different forms of shyness are considered.

Our comparisons of implicit theories of shyness between two experimental shyness conditions provided support for the positivity bias identified in previous North American studies. Prior research by Boseovski and colleagues has shown that the perceived valence of an attribute may influence stability beliefs about that attribute, particularly among children; stronger stability beliefs, or entity views, are more likely to be associated with positive (“good”) attributes than with negative (“bad”) ones (Boseovski, 2010; Heyman and Giles, 2004; Lockhart et al., 2008). Interviews with Chinese children, teachers, and parents have demonstrated that regulated shyness, manifesting as acquiescent, nonassertive, and unassuming behaviors, is often linked to a motivation to fit in with others and is therefore viewed more favorably than anxious shyness, which reflects a motivation to withdraw from social interactions (Xu et al., 2007, 2009, 2020). Similarly, studies of children in North American contexts have found that anxious shyness is associated with low social preference, peer exclusion, and social anxiety, whereas regulated shyness is not (Kong et al., 2023; Xu and Krieg, 2014). Taken together, these findings suggest that regulated shyness is considered a more favorable attribute than anxious shyness in both American and Chinese contexts, which likely contributes to a positivity bias, reflected in stronger entity beliefs about regulated shyness than anxious shyness, regardless of cultural background.

Our expectations regarding variation in relationships with shy peers between American and Chinese children, and across two shyness conditions, were only partially supported. Consistent with our prediction, compared to Chinese children, American children were less likely to engage socially with shy peers regardless of the form of shyness: they reported playing less with shy peers and being more reluctant to form friendships with them. Although American children may view regulated shyness as a more favorable attribute than anxious shyness, the predominant cultural emphasis on assertiveness and sociability likely remains influential and, to some degree, discourages interaction with peers characterized by either form of shyness.

In contrast to our expectation, the two cultural groups did not differ in their liking of shy peers, regardless of the form of shyness. Furthermore, across both cultural groups, although children who read the vignette of regulated shyness expressed greater liking toward the shy peer than those who read the vignette of anxious shyness, they were not more likely to report willingness to play with or befriend the regulated shy peer. Chen (2018) and Chen et al. (2009, 2011) have argued that urban Chinese families have become increasingly susceptible to Western cultural values, and that their traditionally positive views of shyness may have shifted in recent decades. Similarly, Xu and Suh (2022) suggested that being shy, regardless of its form, may place children at a competitive disadvantage in contemporary Chinese society, leading to changing views of shy children, including those displaying regulated shyness, over time. However, these interpretations remain speculative and lack direct empirical support. Future studies are needed to examine how cohort differences in cultural views may be related to evolving attitudes toward, and relationships with, shy peers, particularly regulated shy peers, in the Chinese context.

### The mediating effects of cultural group differences in implicit theories of shyness

As mentioned above, American children on average reported stronger entity beliefs about shyness and poorer relationships with shy peers, raising the possibility that these stronger entity views may, at least to some degree, help explain why they were less willing than Chinese children to play with or befriend shy peers. Our results of the mediation analyses provided empirical support for the potentially important role implicit theories may play in explaining cultural group differences in relationships with shy peers. Previous studies have shown that individual differences in implicit theories of others' attributes significantly influence perceptions, evaluations, and relationships with others (Erdley and Dweck, 1993; Haslam et al., 2004; Levy and Dweck, 1999; Rholes and Ruble, 1984; Yeager and Miu, 2011). Children with stronger entity views of others' attributes are more likely to develop stereotypes and overgeneralizations of behavioral characteristics (Erdley et al., 1997; Levy and Dweck, 1999), and they are less willing to interact with individuals who possess traits they perceive as undesirable (Giles and Heyman, 2004; Levy and Dweck, 1999). Our findings extend prior research by demonstrating similar effects of implicit theories at the group level: that is, a cultural group with stronger entity views may, on average, be less willing to interact with peers characterized by either anxious or regulated shyness.

### The moderating roles of form of shyness

The test of moderated mediation revealed the nuanced nature of the mediating effect of implicit theories of shyness as a function of the form of shyness. Although both American and Chinese children reported stronger entity beliefs about regulated than anxious shyness, possibly due to a positivity bias, the mediating effect of implicit theories of shyness was actually stronger for children who read the vignette depicting anxious shyness. One possible explanation is that strong entity views of a less favorable attribute, such as anxious shyness, may be more detrimental to interpersonal relationships. Children who believe that an unfavorable attribute like anxious shyness is stable and unlikely to change over time may have less patience to play with, or develop friendships with, anxiously shy peers. Thus, compared to entity views of regulated shyness, stronger entity beliefs about anxious shyness among American children may play a more significant role in explaining (i.e., mediating) their poorer relationships with shy peers relative to Chinese children.

### Limitations and future directions

The current study has at least three limitations. First, because our goal was to compare two cultural groups that could not be experimentally manipulated, we employed a quasi-experimental design. Although the two groups were similar in age and gender distribution, they may have differed in other important respects that could confound the observed cultural differences. Consequently, the results of the mediation analyses should not be interpreted as evidence of a causal effect of cultural differences in implicit theories on interpersonal relationships. For example, it is possible that cultural differences in children's relationships with shy peers shape their entity views of anxious and regulated shyness, rather than the reverse.

Second, we focused on implicit theories of others' shyness rather than implicit theories of children's own shyness; the latter may also play a significant role in shaping children's relationships with shy peers. In addition, we did not directly examine whether the two cultural groups differed in other aspects of social cognition, such as their evaluations of or stereotypes about anxious and regulated shyness. Addressing these limitations will be an important direction for future research.

Third, the study was conducted with children recruited from two metropolitan areas: Honolulu and Shanghai. Neither sample is representative of the broader cultural contexts of the United States or China, which limits the generalizability of the findings. Moreover, the unique ethnic composition of the American sample, characterized by a relatively high proportion of children of Asian and multiracial heritage compared to European American heritage, reflects the distinctive demographics of Hawai'i. This composition may have influenced some of the results. For instance, the absence of cultural group differences in liking shy peers may partly reflect the presence of many American children of Asian backgrounds, who may be more likely to experience family socialization practices that encourage modest and unassertive behaviors characteristic of regulated shyness.

Despite these limitations, our findings may have practical implications for education and peer relations. For example, if prior experimental research demonstrating causal links between changes in implicit theories of others' characteristics and adolescents' peer interactions (Yeager et al., 2013a,b) can be replicated in future studies of implicit theories of shyness, school-based interventions aimed at shifting children's entity theories of shyness toward incremental theories may help anxiously or regulated shy children navigate complex and often challenging peer relationships and achieve important developmental milestones, such as gaining social acceptance and forming friendships.

In summary, the current study provides preliminary evidence for the role of implicit theories of anxious (or regulated) shyness in explaining both within-culture individual differences and between-culture group differences in children's relationships with anxiously (or regulated) shy peers. The cultural similarities and differences observed here may also lay the groundwork for further research on the links between interpersonal perceptions of different forms of shyness and the socioemotional adjustment of anxiously shy and regulated shy children across cultural contexts.

## Data Availability

The raw data supporting the conclusions of this article will be made available by the authors, without undue reservation.
