# Supplementary material for: Implicit theories of anxious and regulated shyness in American and Chinese children
Source: Front Psychol. 2026 Mar 17;17:1766694. doi: 10.3389/fpsyg.2026.1766694 (PMC13035493; doi:10.3389/fpsyg.2026.1766694)
Supplement: Supplementary file 1 [file Table_1.docx]

**Supplementary Materials**

**1. Approximate measurement invariance analyses**

The alignment optimization approach was used to examine approximate measurement invariance of implicit theories of shyness. The results of configural invariance demonstrated support for configural invariance (i.e., same one-factor structure of entity theories for both groups; *χ*^2^(10) = 8.19, *p* = .61, CFI = 1.00, TLI = 1.00, RMSEA = .04, 90%CI[.00, .09], *p* (RMSEA ≤ .05) = .77, SRMR = .02). The results of alignment optimization indicated that approximate measurement invariance held for all the item loadings and intercepts across groups. In addition, American children (the reference group; latent factor mean = 0) reported higher entity theories of shyness than Chinese children (latent factor mean = -.739).

The alignment optimization approach was also used to examine approximate measurement invariance of the anxious and regulated shyness. The results of configural invariance demonstrated support for configural (i.e., same two-factor structure for both groups; *χ*^2^(68) = 75.74, *p* = .24, CFI = .99, TLI = .99, RMSEA = .03, 90%CI[.00, .07], *p* (RMSEA ≤ .05) = .73, SRMR = .06). The results of alignment optimization indicated that approximate measurement invariance held for all the item loadings and intercepts across groups. In addition, Chinese children (latent factor mean = .625) were rated by their parents as having higher regulated shyness than American children (reference group, latent factor mean = 0), But the two groups did not differ in anxious shyness (latent factor mean = .07 and 0, respectively).

**2. Sensitivity analyses regarding ethnic subgroups**

Given that the participants of American children included several ethnic subgroups, we conducted sensitivity analyses (e.g., repeating analyses while excluding one subgroup at a time) to demonstrate the robustness of the moderated mediating effect. When excluding Native Hawaiian and Pacific Islanders (11.7% of the American sample), we replicated the same moderated mediation effect, with an estimated Index of Moderated Mediating (IMM) effect of .12 (*p* = .01; 95%CI[.02, .21]). When excluding European Americans (13.8% of the American sample), we also replicated the same moderated mediation effect, with an estimated Index of Moderated Mediating (IMM) effect of .12 (*p* = .01; 95%CI[.02, .21]). When excluding multiracial children (35.1% of the American sample), the estimated Index of Moderated Mediating (IMM) effect was .11 (*p* = .03; 95%CI[.01, .21]). When excluding Asian American children (39.4% of the American sample), the estimated Index of Moderated Mediating (IMM) effect was .14 (*p* = .01; 95%CI[.03, .25]). Thus, it appears that the moderated mediating effect of shyness condition was relatively robust.
